# Supplementary material for: Plug & play origami modules with all-purpose deformation modes
Source: Nat Commun. 2023 Jul 19;14:4329. doi: 10.1038/s41467-023-39980-7 (PMC10356792; doi:10.1038/s41467-023-39980-7)
Supplement: Supplementary file 1 — Supplementary Information [file 41467_2023_39980_MOESM1_ESM.pdf]

Supplementary Information for

Plug & Play Origami Modules with All-Purpose Deformation Modes

Chao Zhang, Zhuang Zhang, Yun Peng, Yanlin Zhang, Siqi An, Yunjie Wang,

Zirui Zhai, Yan Xu, and Hanqing Jiang

Correspondence to: Hanqing Jiang, hanqing.jiang@westlake.edu.cn
Yan Xu, xyzs@zju.edu.cn

This PDF file includes:

Supplementary Note 1. Deformation characterization.
Supplementary Note 2. Analysis of the influence of P_p on H.
Supplementary Note 3. Output force
Supplementary Note 4. Details in experiments of the robotic arm.
Supplementary Note 5. Calculation of the working boundary of the robotic arm.
Supplementary Fig. 1. Geometry of the one-level Kresling structure.
Supplementary Fig. 2. Experimental and simulated results of the compression testing.
Supplementary Fig. 3. Fabrication process of the present origami actuator.
Supplementary Fig. 4. Experimental images of the fabrication process.
Supplementary Fig. 5. Inner details of the present origami module.
Supplementary Fig. 6. Geometry of the origami main chamber.
Supplementary Fig. 7. Simulation results of the twisting-contraction motion and the comparison among the experimental, analytical, and FEA results.
Supplementary Fig. 8. Simulation results of the bending-twisting motion and the comparison among the experimental and FEA results.
Supplementary Fig. 9. Measurement of the height H, bending angle α, and twisting angle β.
Supplementary Fig. 10. Simulation results of the contraction motion and the comparison between the experimental and FEA results.
Supplementary Fig. 11. Simulation results of the bending motion and the comparison between the experimental and FEA results.
Supplementary Fig. 12. Simulation results of the twisting motion and the comparison between the experimental and FEA results.

Supplementary Fig. 13. Simulation results of the twisting-contraction motion and the comparison between the experimental and FEA results.
Supplementary Fig. 14. Simulation results of the bending-twisting motion and the comparison between the experimental and FEA results.
Supplementary Fig. 15. Simulation results of the bending-contraction motion and the comparison between the experimental and FEA results.
Supplementary Fig. 16. The relationship between the height *H* and the negative pressure value in the main chamber, under different input positive pressure values inside the pouches.
Supplementary Fig. 17. Simulation results of the bending-contraction motion with P_p = 45 kPa.
Supplementary Fig. 18. Simulation results of the bending-contraction motion with P_p = 65 kPa.
Supplementary Fig. 19. Simulation results of the bending-twisting-contraction motion and the comparison between the experimental and FEA results.
Supplementary Fig. 20. Measurements of the output contraction force (a), bending force (b), and twisting moment (c).
Supplementary Fig. 21. Working space of the 3-module and 4-module of the origami robotic arm.
Supplementary Fig. 22. Fabrication process of the vacuum-actuated gripper.
Supplementary Fig. 23. Control system of the three-module/four-module robotic arm.
Supplementary Fig. 24. Three-module robotic arm.
Supplementary Fig. 25. Deformation of a module with different deflation speed in contraction (a) and bending motion (b).
Supplementary Tab. 1. Parameters of the Equation S1 and Equation S2.
Supplementary Tab. 2. Parameters of the Equation S3 and Equation S4.
Supplementary Tab. 3. Parameters of the Equation S5 and Equation S6.

Supplementary Note 1. Deformation characterization

We measured the deformation of the origami modules on a testing platform (see Supplementary Movie 1 - Supplementary Movie 9). The initial pressure in pouches and the main chamber of the origami module in each experiment is the same as the external atmosphere. The bottom plate of the origami is fixed on the platform, then we actuate the side pouches with positive pressure. During the actuation process, the main chamber is vacuumized after the positive pressure inside the pouches reaches its prescribed value. The pressure values are regulated by precision regulators (IRV20-C06, SMC for positive pressure, and IR1000-01-A, SMC for vacuum). The deformation of the origami is recorded by cameras on three different views (front, side, and top views). The bending angle, twisting angle, and height are defined in Supplementary Fig. 9 and are measured in Tracker 4.91. It should be noted that the height is characterized by the arc length (i.e., contour height) on the tensile side (marked as the solid red line in Supplementary Fig. 9b, when the side pouches are actuated. If the side pouches are deflated, the height is the length of a straight line, as shown in Supplementary Fig. 9d. To be specific, in Fig. 3b, Fig. 3d and Fig. 3e, the height H is the length of the straight line between the top and bottom cap. In Fig. 3c and Fig. 3g, the height H is the arc length between the top and bottom cap. In Fig. 3f, the height H is defined as that in Supplementary Fig. 9b. In Fig. 3h, the height H is defined as that in Supplementary Fig. 9d.

Supplementary Note 2. Analysis of the influence of P_p on H

We utilize the finite element method to analyze the influence of different P_p on H**,** and the results are shown in Supplementary Figs. 15 – 18. With the increment of P_p from 20 to 90 kPa, H increases under the same P_m. It can also be seen that H change less during the whole deformation process with the augment of P_p. When the P_p reaches 90 kPa, the change of H is about 2.3%, which is negligible.

Supplementary Note 3. Output force

The measurements and results of the output contraction force, bending force, and twisting moment are shown in Supplementary Fig. 20. The force sensor (Mini 45, ATI Industrial Automation Co., LTD, USA) is fixed on a fixed beam. The bottom of the origami module is fixed on the platform, and the top of the module is connected to the force sensor via a plastic bolt. During the contraction process (Supplementary Fig. 20a), the tension force F_c increases with the increase of the vacuum value, and the maximum force is 22.9 N (when P_m = -8 kPa). As to the bending process (Supplementary Fig. 20b), the shearing force F_b also becomes larger with the increase of the vacuum value in the main chamber; the maximum value of F_b is 1.91 N (when P_m = -12 kPa). In the first stage (pressurizing bottom pouches) of the twisting motion (Supplementary Fig. 20c), the torque is along the negative direction of the Z-axis. However, in the second and third stages, the direction of the torque changes to the positive direction of the Z-axis. The maximum value of T_t is 0.21 Nm.

Supplementary Note 4. Details in experiments of the robotic arm

The 3-module robotic arm consists of three origami modules and a vacuum-actuated gripper (Fig. 4a). The thin pneumatic tubes, through which the fluid (air or water) is pumped in or exhausted out of pouches, are placed in the main chamber. The proximal end of the robotic arm is fixed; the rest is free to generate extension / contraction, bending, twisting, and coupled deformation under pressurization. The motion of the three-module / four-module robotic arm is recorded in two different views, namely the front and top views.

The connection between every two adjacent parts is the bolt-nut connection, as shown in Fig. 4a. The plug & play process (see Fig. 4d and Supplementary Movie 13) can be achieved as follows: Remove the original v-shape groove and air sealing layer, followed by replacing another v-shape groove and the corresponding air sealing layer. Then the fourth module is screwed on the 3-module robotic arm to form a 4-module robotic arm.

Supplementary Note 5. Calculation of the working boundary of the robotic arm

In order to calculate the working boundaries (Fig. 4d and Supplementary Fig. 21) of the 3-module and 4-module robotic arms, we obtain the relationship between bending angle γ and α, as well as the relationship between γ and H' in experiments, when P_p = 90 kPa (Supplementary Fig. 24d and Supplementary Fig. 24e), P_p = 20 kPa (Supplementary Fig. 21a) and P_p = 65 kPa (Supplementary Fig. 21b). The results are shown in the following:

$$H' = P_1\gamma^5 + P_2\gamma^4 + P_3\gamma^3 + P_4\gamma^2 + P_5\gamma + P_6 \tag{S1}$$

$$\alpha = P_7\gamma + P_8 \tag{S2}$$

the parameters P_1- P_8 are shown in Supplementary Table 1.

$$H' = Q_1\gamma^5 + Q_2\gamma^4 + Q_3\gamma^3 + Q_4\gamma^2 + Q_5\gamma + Q_6 \tag{S3}$$

$$\alpha = Q_7\gamma^5 + Q_8\gamma^4 + Q_9\gamma^3 + Q_{10}\gamma^2 + Q_{11}\gamma + Q_{12} \tag{S4}$$

the parameters Q_1- Q_{12} are shown in Supplementary Table 2.

$$H' = S_1\gamma^5 + S_2\gamma^4 + S_3\gamma^3 + S_4\gamma^2 + S_5\gamma + S_6 \tag{S5}$$

$$\alpha = S_7\gamma^3 + S_8\gamma^2 + S_9\gamma + S_{10} \tag{S6}$$

the parameters S_1- S_{10} are shown in Supplementary Table 3.

Conventional Kresling actuator can only contract and twist along its axial direction, making its workspace a single straight line, as shown in Supplementary Fig. 21c and Supplementary Fig. 21d (orange line). However, after selectively breaking the energy favorable deformation mode of the Kresling origami pattern, the actuator we obtained can exhibit bidirectional bending (the design with two side pouches), leading to an expansion of its workspace from a straight line to a planar area (irregular polygon ADE

in Supplementary Fig. 21c). Besides, after the 3-module robotic arm is assembled with another module forming a 4-module robotic arm, its workspace can be further expanded (irregular polygon FLM in Supplementary Fig. 21d). The black solid lines, red dash-dot lines and blue dash-dot lines (in Supplementary Fig. 21c and Supplementary Fig. 21d) can be calculated as the following equations (Eq. S7 and Eq. S8). The green solid lines in Supplementary Fig. 21c and Supplementary Fig. 21d are fitted with the points A, B, C and D as well as F, G, J and L, respectively.

$$x = -\left(\sum_{i=0}^{m-1} \left[H' \cos\left(\gamma + i\alpha\right) \right] + \left|BC\right| \cos\left(m\alpha\right) \right) \tag{S7}$$

$$y = \sum_{i=0}^{m-1} \left[H' \sin\left(\gamma + i\alpha\right) \right] + \left|BC\right| \sin\left(m\alpha\right) \tag{S8}$$

where *m* represents the amount of bending module. The analysis of Fig. 4e does not consider the planar bending of the distal module, in accordance with the real working status (pouring water from the cup) of the prototyped robotic arm. Note that the analysis in this note is based on the design with two side pouches integrated inside each origami module for the sake of ease of fabrication. If all six sides of the present origami module are integrated with pouches, omnidirectional movement can be achieved, then forming a spatial workspace (the current planar one spanning around the axial direction).

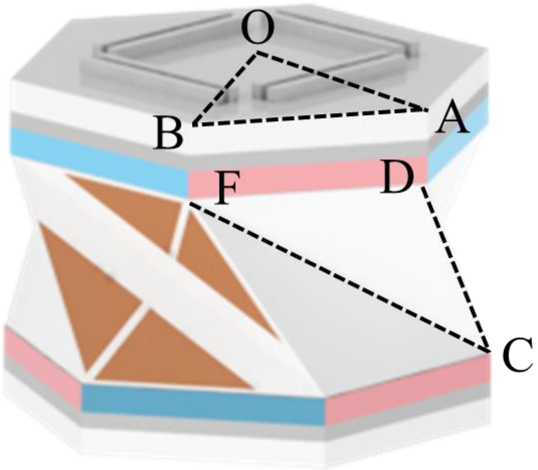

Supplementary Fig. 1. Geometry of the one-level Kresling structure. λ is the ratio between the angle $\angle FCD$ **and** $\angle OAB$**.**

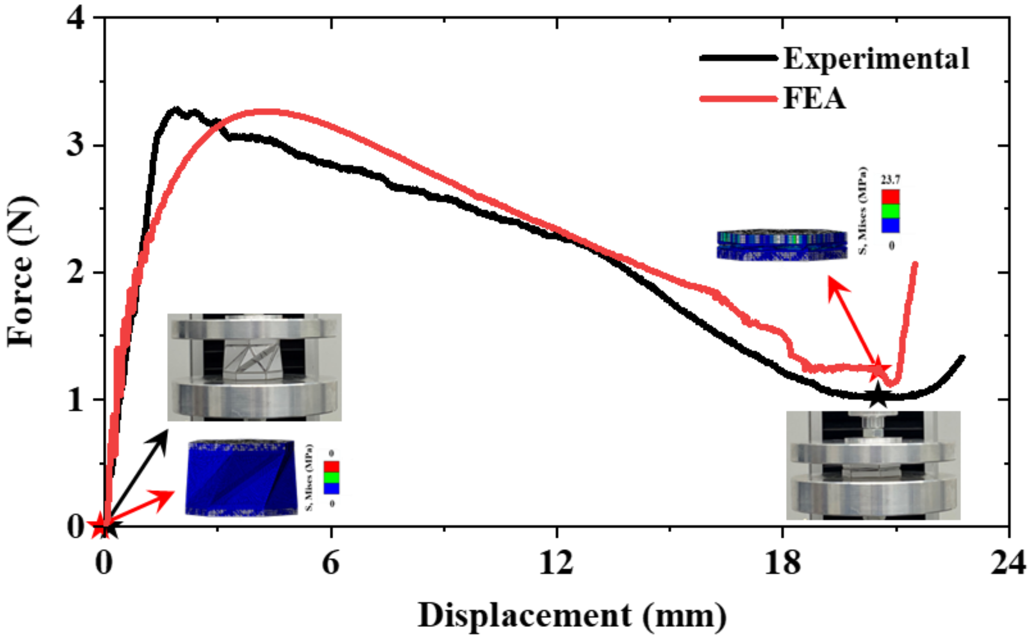

Supplementary Fig. 2. Experimental and simulated results of the compression testing. The black and red solid lines indicate the experimental and simulation results respectively.

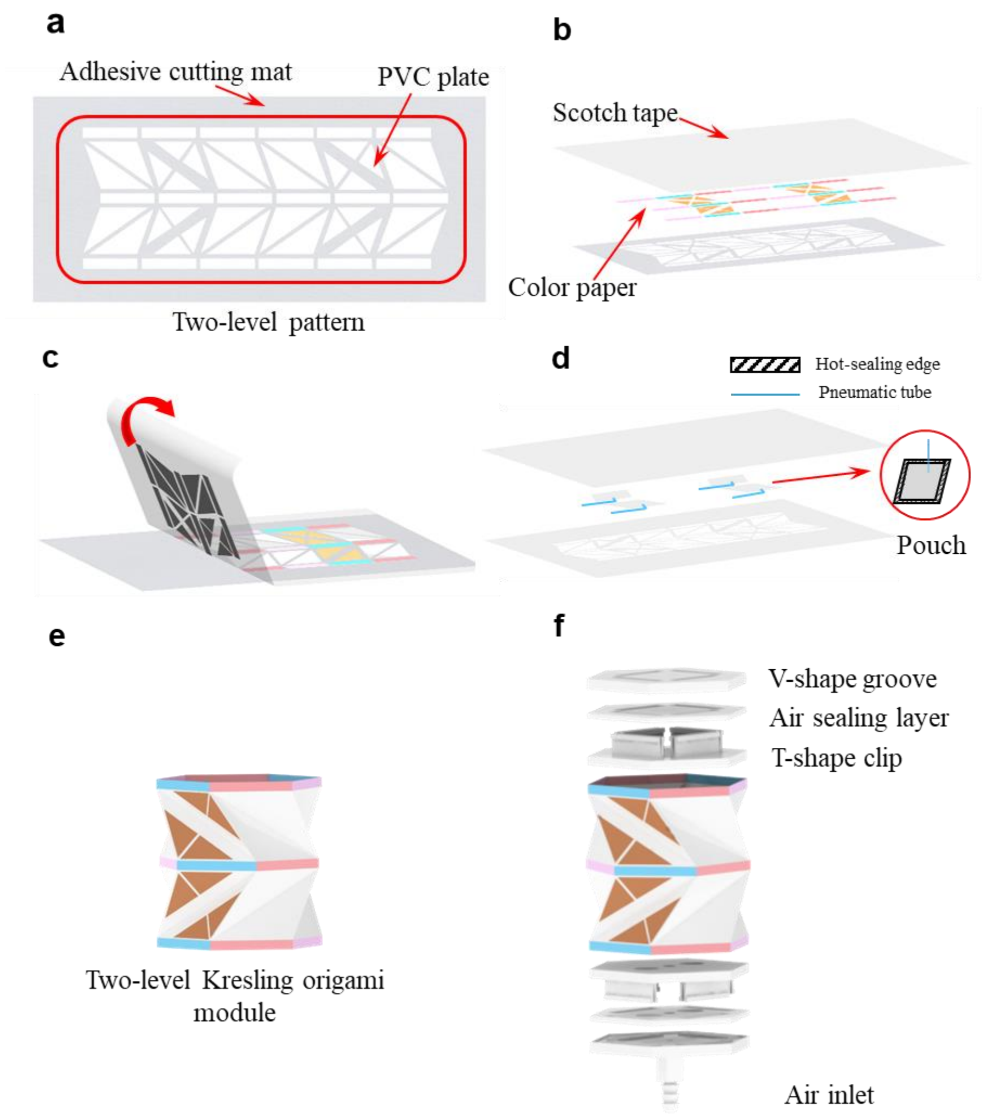

Supplementary Fig. 3. Fabrication process of the present origami actuator. a, Step 1: the Kresling patterns (one level or two levels) made of PVC panels are cut on adhesive cutting mats using Silhouette CAMEO 4; **b,** Step 2: color papers are attached on those PVC panels, followed by covering a piece of scotch tap; **c,** Step 3: the color paper and the PVC panels are all peeled off from the adhesive cutting mats by uncovering the scotch tap; **d,** Step 4: a soft thin tube is stuck into the pouch, followed by sealing the sides of pouches with a heat sealing machine (Deli 16499, Deli Group Co., Ltd, China), and the four pouches are attached on the back side of the four two-state cell, a piece of scotch tap is covered on the pouches, PVC panels and the scotch tap; **e,** Step 5: a two-level pattern is formed by rolling this sandwiching structures; **f,** Step 6: the V-shape groove, Ecoflex layer for air sealing and T-shape clips are put on the top and bottom ends of the two-level pattern.

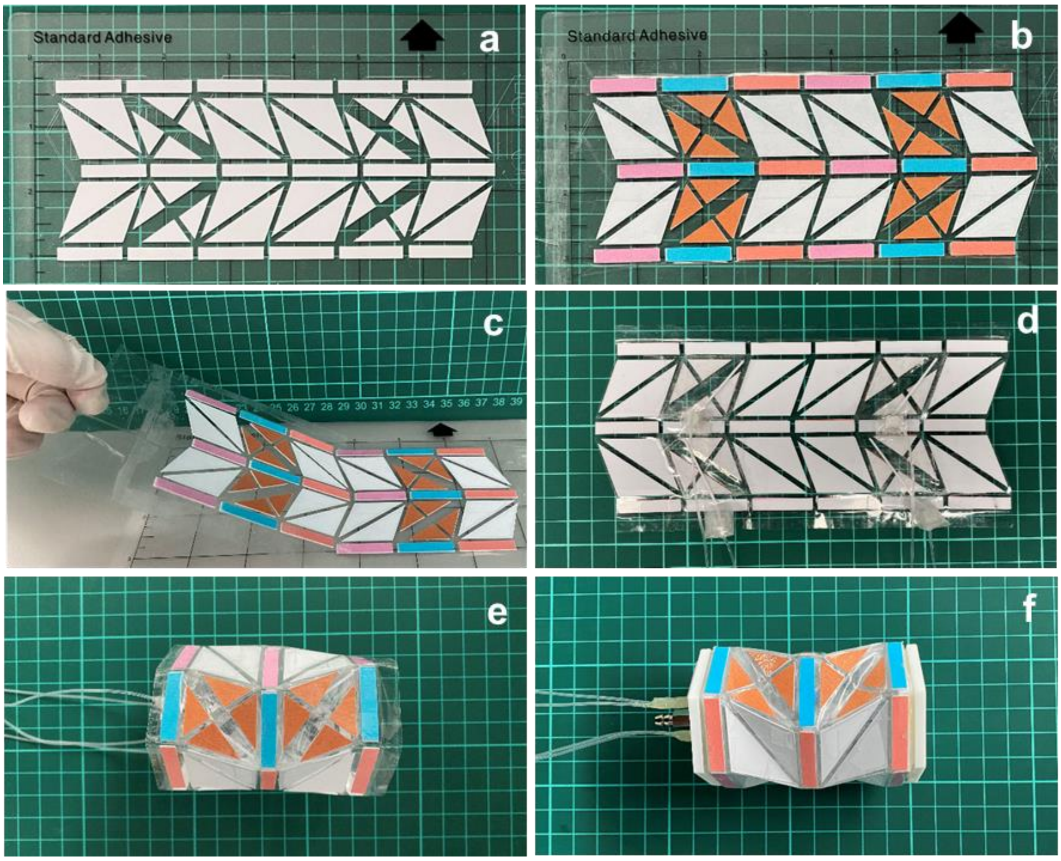

Supplementary Fig. 4. Experimental images of the fabrication process. (**a**-**f**) correspond to steps 1-6 (**a**-**f**) in Supplementary Fig. 1.

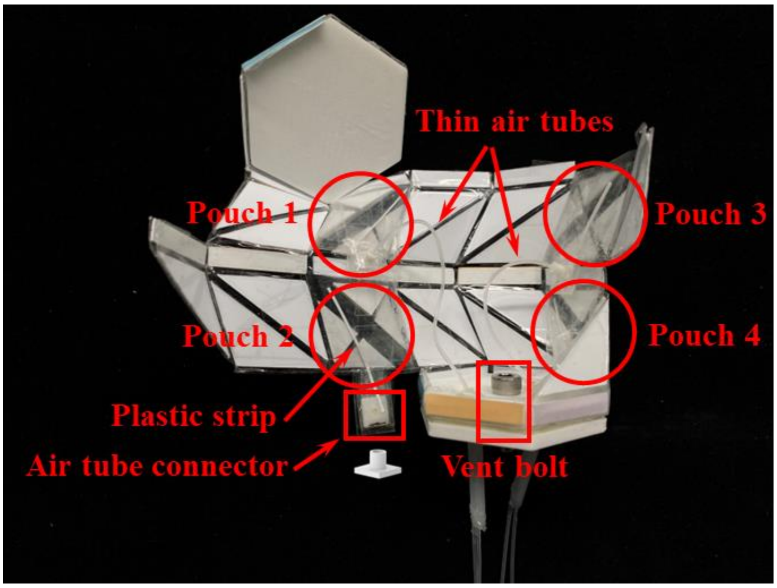

Supplementary Fig. 5. Inner details of the present origami module. Each pouch consists of a TPU bag, a plastic strip, an air tube connector and a thin air tube. The plastic strip is utilized to maintain the air path when the pouch is folded. The air tube connector is used to link the air tube and the TPU bag. All the soft air tubes are placed in the main chamber. We inflate/deflate pouches through air tubes and deflate the main chamber via the vent bolt. Each pouch is linked with two solenoid valves (one for positive pressure control (V114-5LZB, SMC Inc.) and the other for vacuum control (VK332V-5G-01, SMC Inc.)) through thin air tubes. The pressure inside pouches is controlled by precision regulators (IRV20-C06, SMC Inc.). The main chamber is actuated by vacuum with a precision regulator (IR1000-01-A, SMC Inc.).

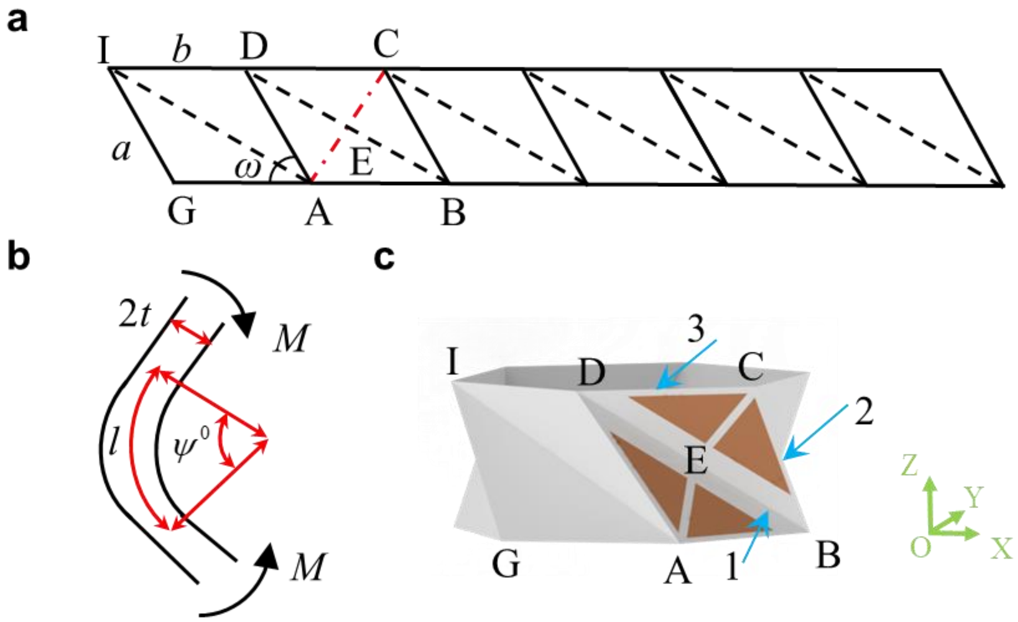

Supplementary Fig. 6. Geometry of the origami main chamber. a, The one-level pattern. The dotted lines represent valley creases, and the dash-dot lines indicate the added creases, a, b, α represent the length of IG, ID and the angle of between AG and AD, respectively. **b,** The origami structure under a deployed configuration. 1, 2 and 3 represent three types of creases, respectively. **c,** Geometric parameters of a crease. $2t$, l and ψ^0 are the thickness, width, and initial angle of a crease.

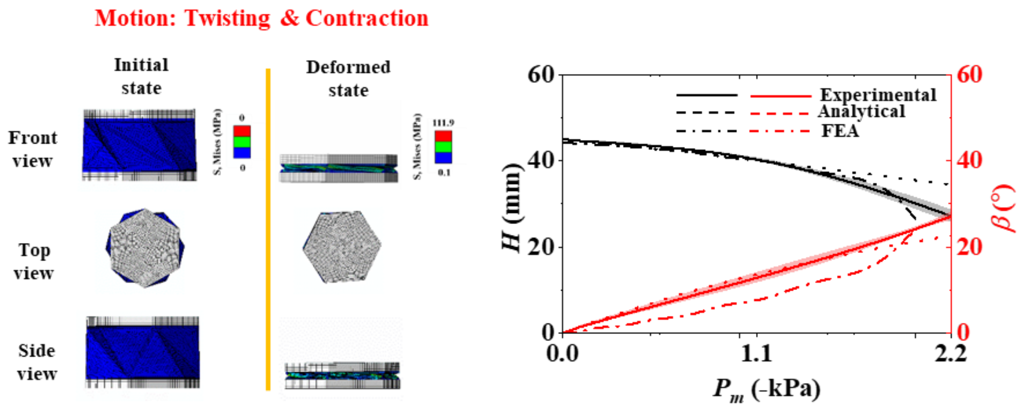

Supplementary Fig. 7. Simulation results of the twisting-contraction motion and the comparison among the experimental, analytical, and FEA results. The black solid, dashed and dashed-dotted lines represent the experimental, analytical and FEA results between the H and P_m. The red solid, dashed and dashed-dotted lines indicate the experimental, analytical and FEA results between the β and P_m. The left part of the figure gives the initial state and the deformed state of the finite element model in front view, top view and side view.

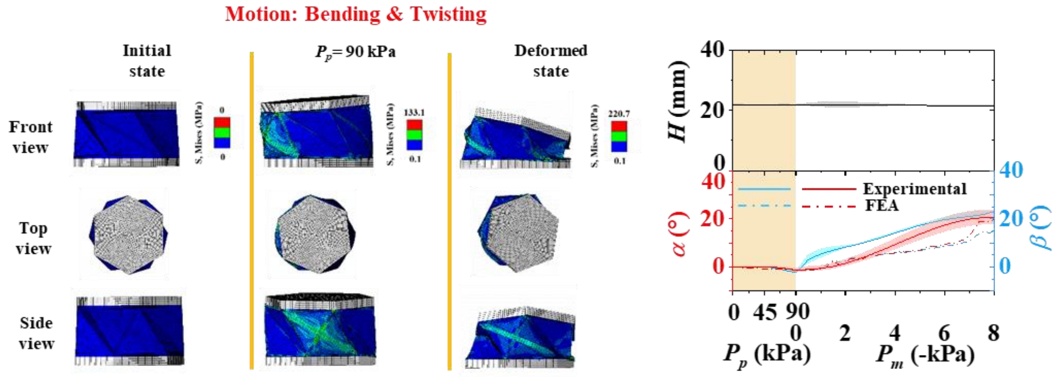

Supplementary Fig. 8. Simulation results of the bending-twisting motion and the comparison between the experimental and FEA results. The red solid and dashed-dotted lines represent the experimental and FEA results between the α and P_m (P_p). The blue solid and dashed-dotted lines indicate the experimental and FEA results between the β and P_m (P_p). The left part of the figure gives the initial state and the deformed state of the finite element model in front view, top view and side view.

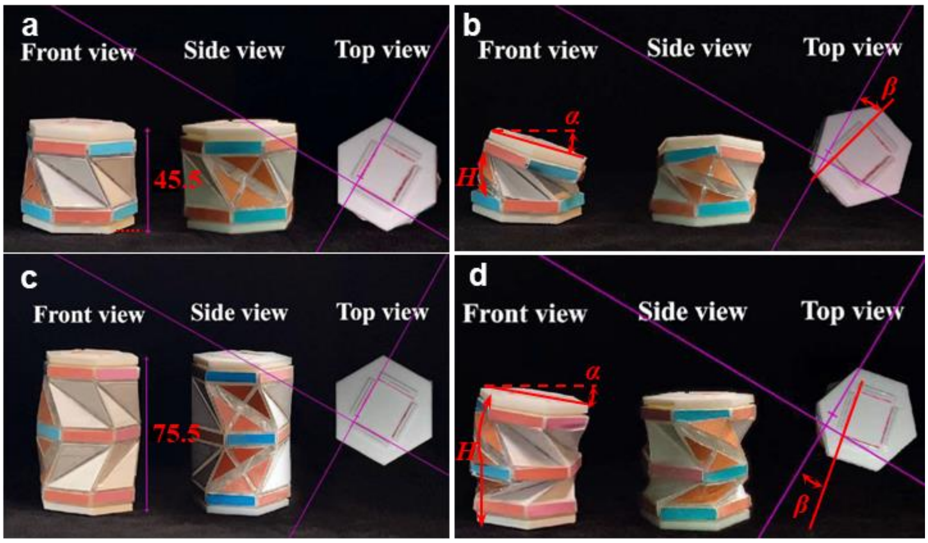

Supplementary Fig. 9. Measurement of the height *H*, bending angle *α*, and twisting angle *β*. Initial state of one-level **(a)** / two-level **(c)** origami structures in front, side and top view. **b,** Deformed state of the one-level origami structure. The bending angle *α* and pouch height *H* of the one-level origami structure are measured in the front view. We define the changing angle of the top cap as the bending angle *α*. The arc length is defined as the height *H* (in **Fig. 2c**). Twisting angle *β* is measured in the top view. We define the changing angle of the red line on the top cap as the twisting angle. **d,** Deformed state of the two-level origami structure. The bending angle *α* and twisting angle *β* are defined as the counterparts in Supplementary Fig. 18b.

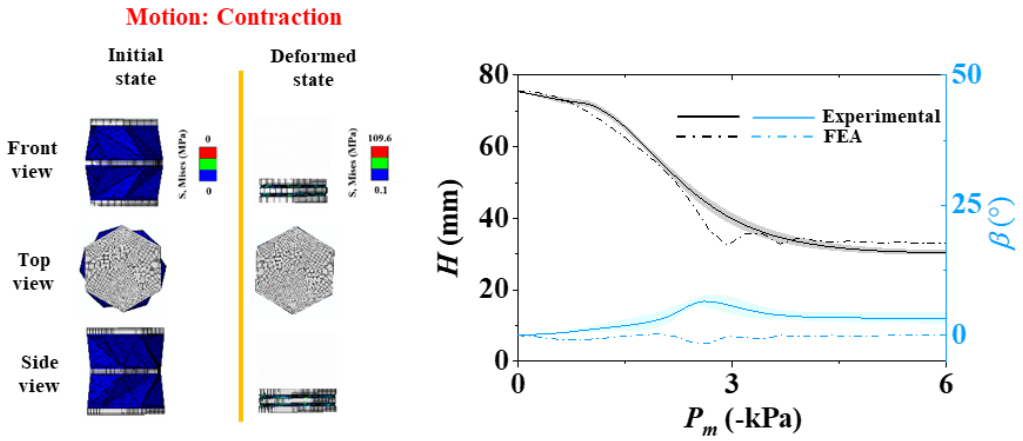

Supplementary Fig. 10. Simulation results of the contraction motion and the comparison between the experimental and FEA results. The black solid and dashed-dotted lines represent the experimental and FEA results between the H and P_m. The blue solid and dashed-dotted lines indicate the experimental and FEA results between the β and P_m. The left part of the figure gives the initial state and the deformed state of the finite element model in front view, top view and side view.

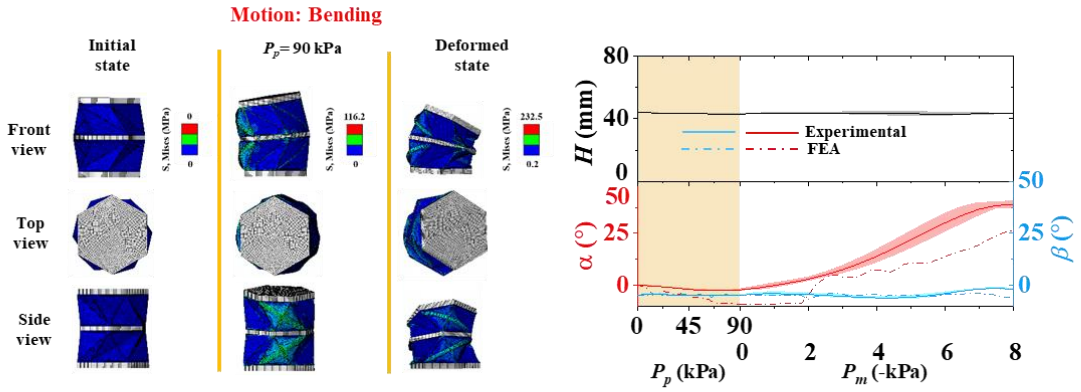

Supplementary Fig. 11. Simulation results of the bending motion and the comparison between the experimental and FEA results. The red solid and dashed-dotted lines represent the experimental and FEA results between the α and P_m (P_p). The blue solid and dashed-dotted lines indicate the experimental and FEA results between the β and P_m (P_p). The left part of the figure gives the initial state and the deformed state of the finite element model in front view, top view and side view.

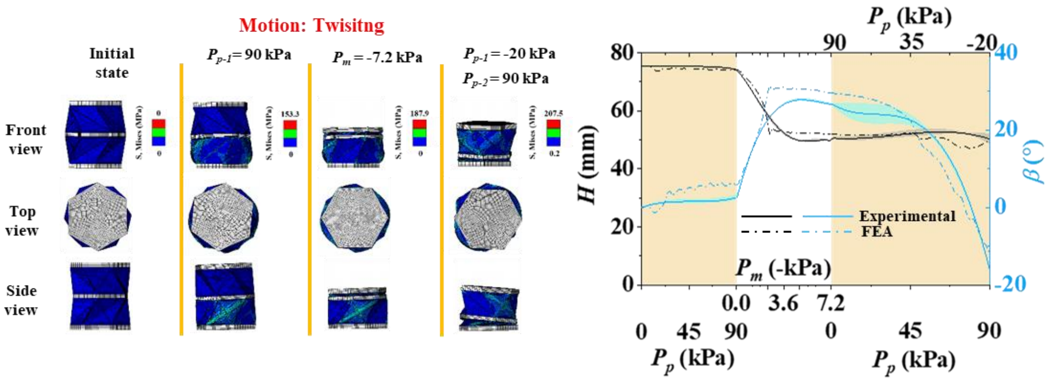

Supplementary Fig. 12. Simulation results of the twisting motion and the comparison between the experimental and FEA results. The black solid and dashed-dotted lines represent the experimental and FEA results between the H and P_m (P_p). The blue solid and dashed-dotted lines indicate the experimental and FEA results between the β and P_m (P_p). The left part of the figure gives the initial state and the deformed state of the finite element model in front view, top view and side view.

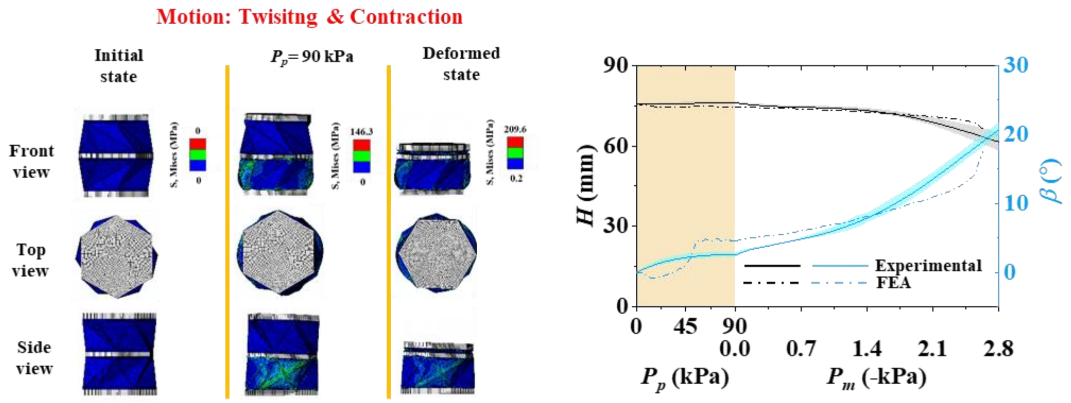

Supplementary Fig. 13. Simulation results of the twisting-contraction motion and the comparison between the experimental and FEA results. The black solid and dashed-dotted lines represent the experimental and FEA results between the H and P_m (P_p). The blue solid and dashed-dotted lines indicate the experimental and FEA results between the β and P_m (P_p). The left part of the figure gives the initial state and the deformed state of the finite element model in front view, top view and side view.

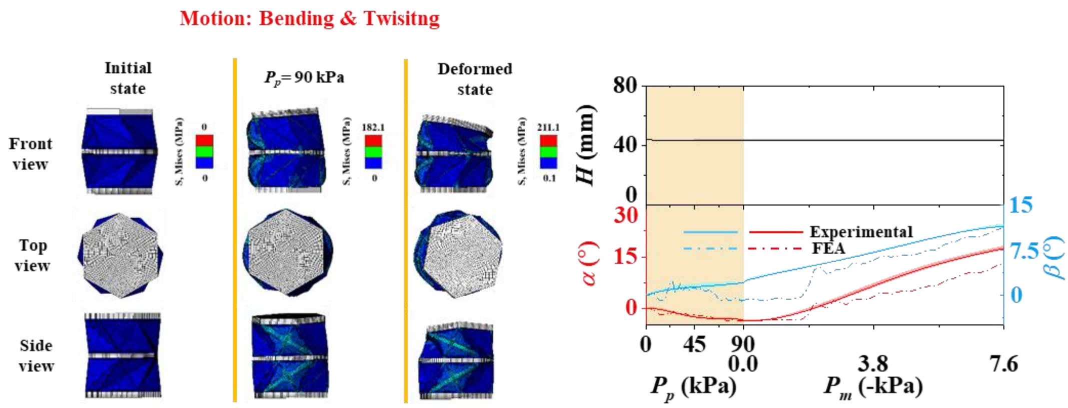

Supplementary Fig. 14. Simulation results of the bending-twisting motion and the comparison between the experimental and FEA results. The red solid and dashed-dotted lines represent the experimental and FEA results between the α and P_m (P_p). The blue solid and dashed-dotted lines indicate the experimental and FEA results between the β and P_m (P_p). The left part of the figure gives the initial state and the deformed state of the finite element model in front view, top view and side view.

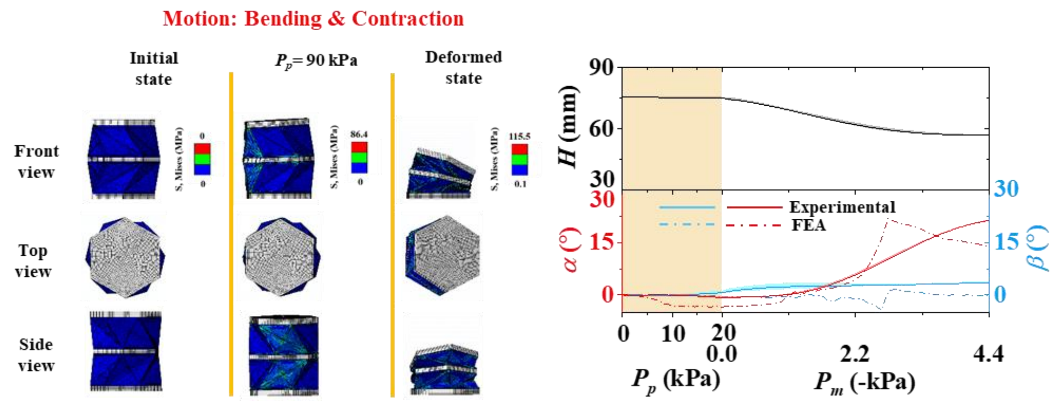

Supplementary Fig. 15. Simulation results of the bending-contraction motion and the comparison between the experimental and FEA results. The red solid and dashed-dotted lines represent the experimental and FEA results between the α and P_m (P_p). The blue solid and dashed-dotted lines indicate the experimental and FEA results between the β and P_m (P_p). The left part of the figure gives the initial state and the deformed state of the finite element model in front view, top view and side view.

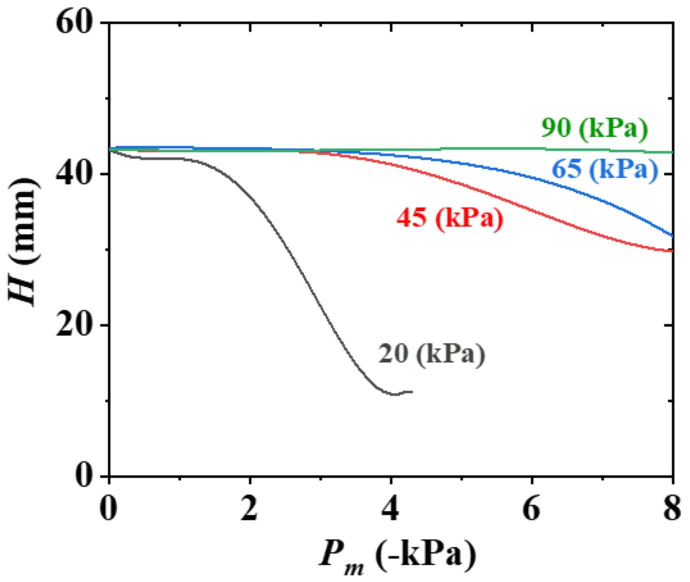

Supplementary Fig. 16. The relationship between the height and the negative pressure value in the main chamber, under different input positive pressure values inside the pouches. The green, blue, red, and black solid lines represent the relationship between H and P_m when P_p = 90, 65, 45, and 20 kPa.

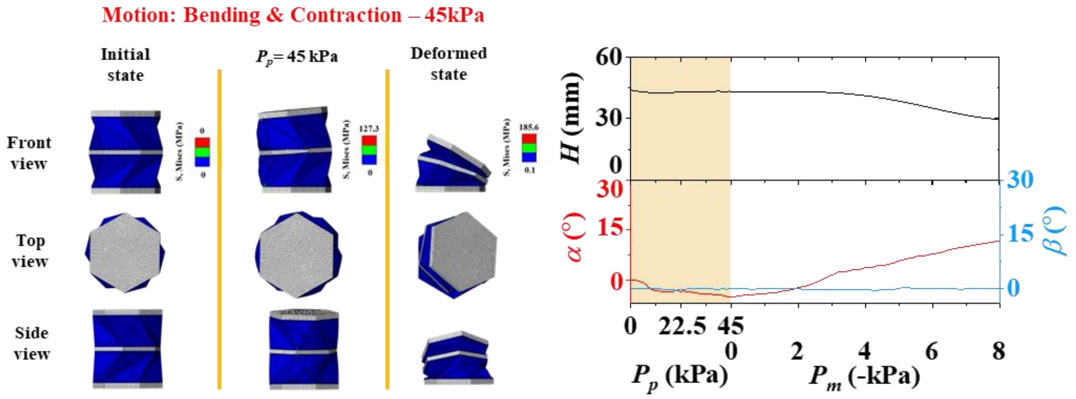

Supplementary Fig. 17. Simulation results of the bending-contraction motion with P_p = 45 kPa. The black, red and blue solid lines represent the relationship between H and P_m (P_p), α and P_m (P_p), β and P_m (P_p), respectively.

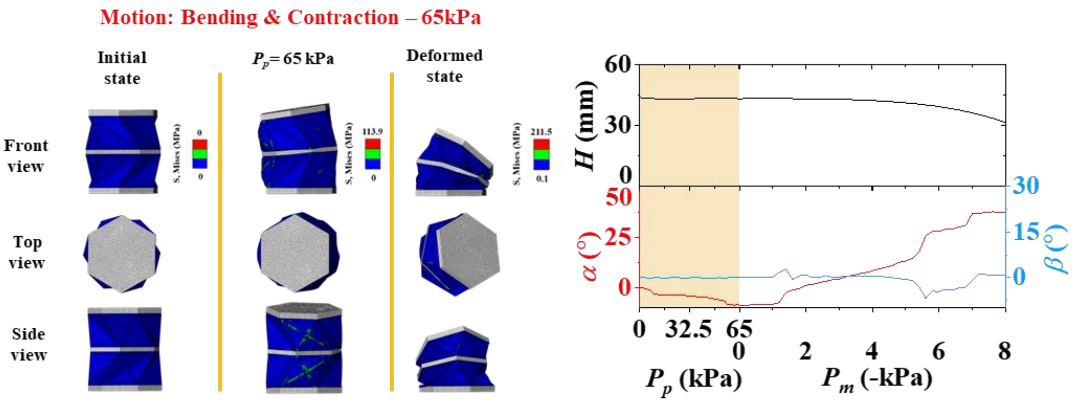

Supplementary Fig. 18. Simulation results of the bending-contraction motion with P_p = 65 kPa. The black, red and blue solid lines represent the relationship between H and P_m (P_p), α and P_m (P_p), β and P_m (P_p), respectively.

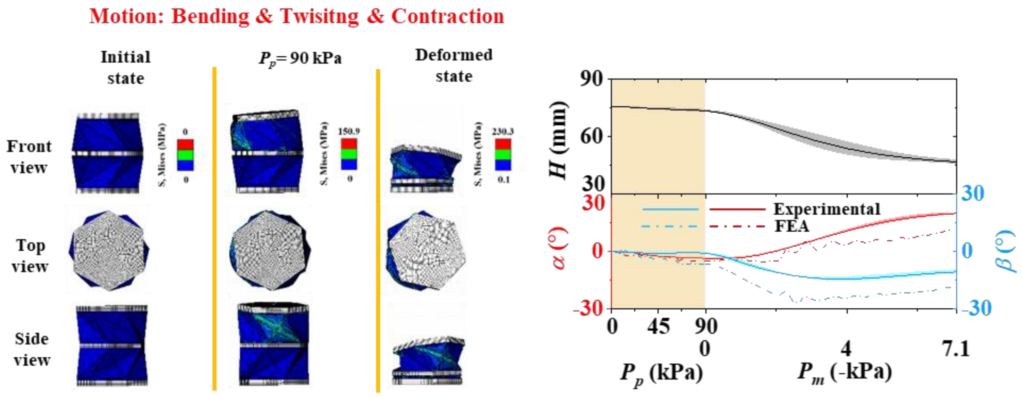

Supplementary Fig. 19. Simulation results of the bending-twisting-contraction motion and the comparison between the experimental and FEA results. The red solid and dashed-dotted lines represent the experimental and FEA results between the α and P_m (P_p). The blue solid and dashed-dotted lines indicate the experimental and FEA results between the β and P_m (P_p). The left part of the figure gives the initial state and the deformed state of the finite element model in front view, top view and side view.

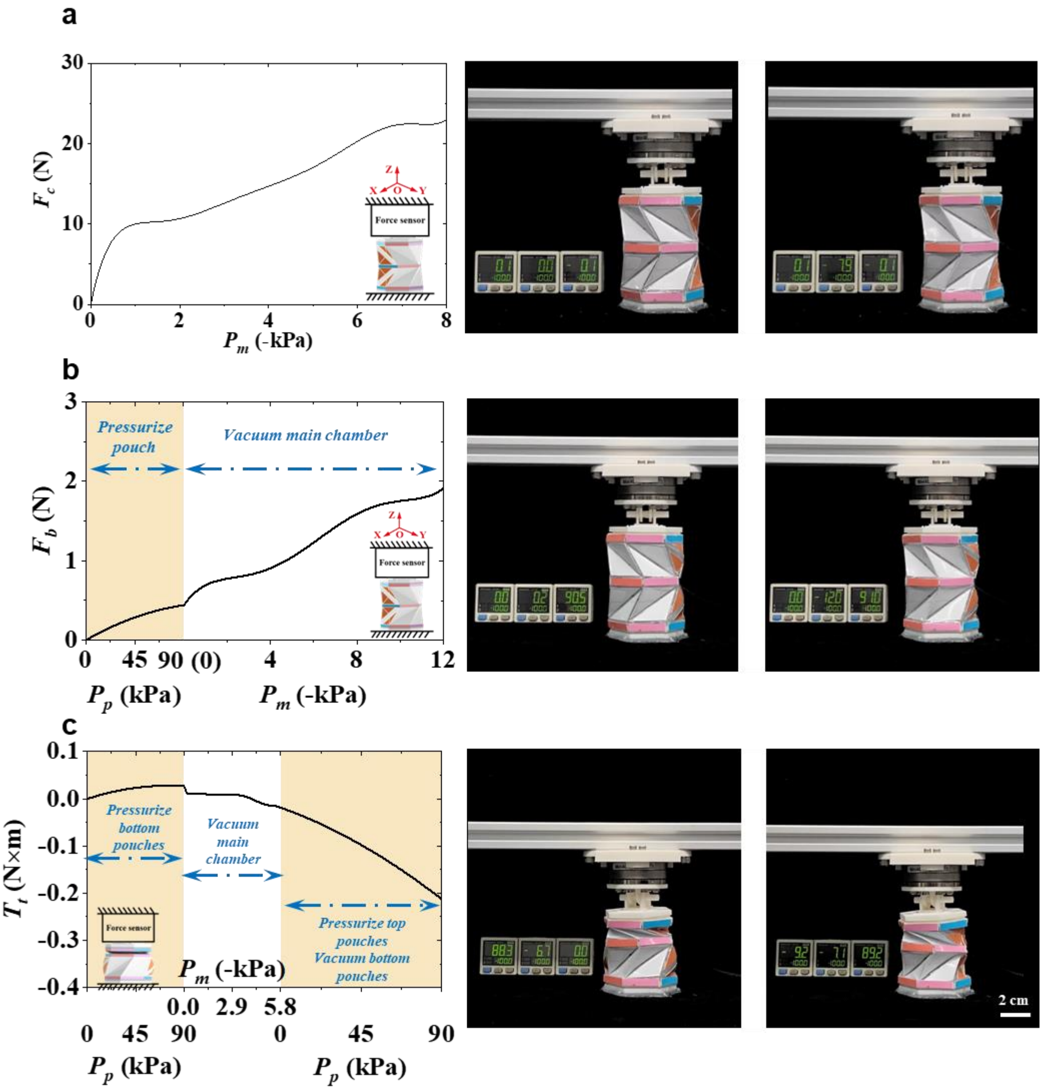

Supplementary Fig. 20. Measurements of the output contraction force (a), bending force (b), and twisting moment (c). F_c and F_b are the force along the Z-axis and X-axis, respectively. T_t is the torque along the Z-axis.

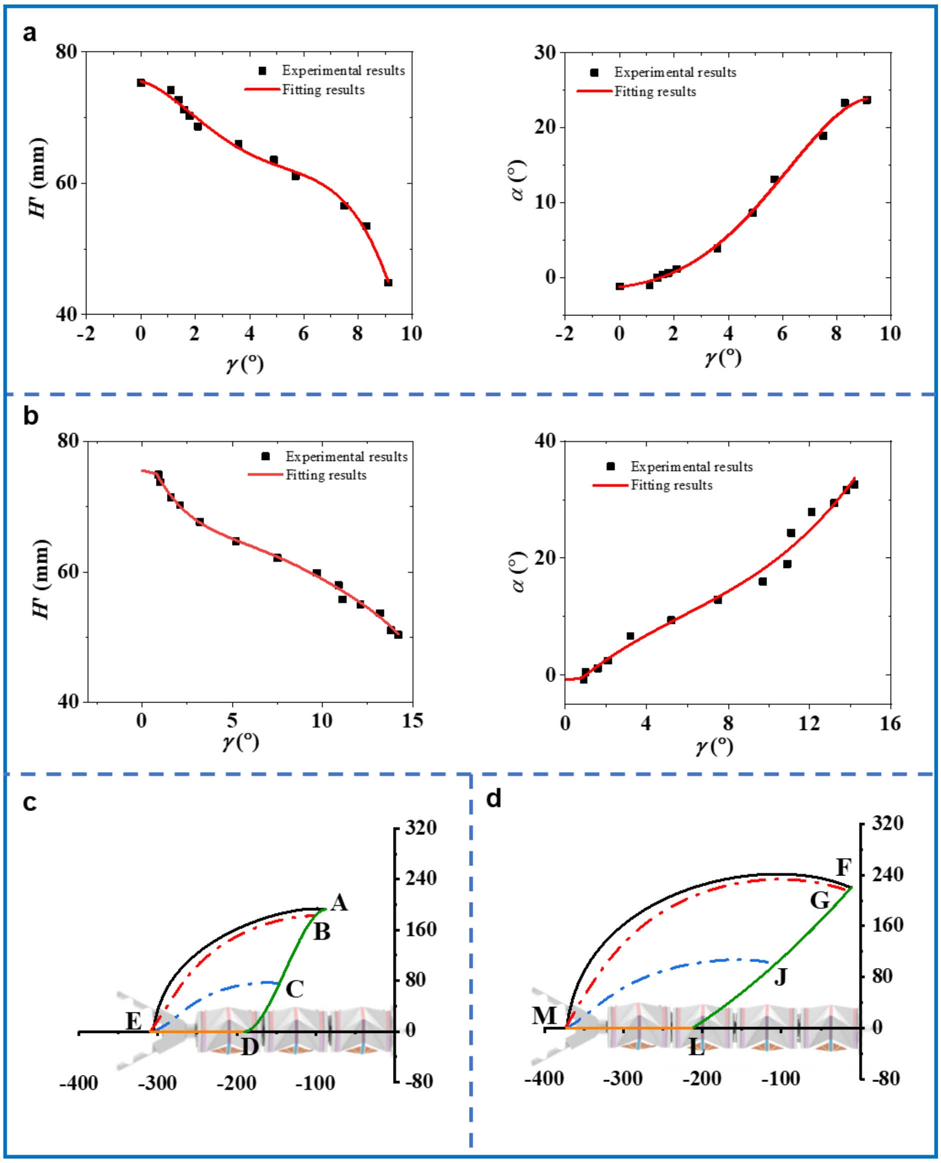

Supplementary Fig. 21. Workspace of the 3-module and 4-module robotic arms. The relationship between the bending angles γ and α, as well as the relationship between γ and H' in the condition of P_p = 20 kPa (**a**) and P_p = 65 kPa (**b**). The workspace of the 3-module (**c**) and 4-module (**d**) robotic arms. The black solid lines, red dash-dot lines and blue dash-dot lines are the working boundaries of the 3/4 module arms bending in the condition of P_p = 90 kPa, P_p = 65 kPa and P_p = 20 kPa, respectively. The orange solid lines are the working boundaries when the arms contract.

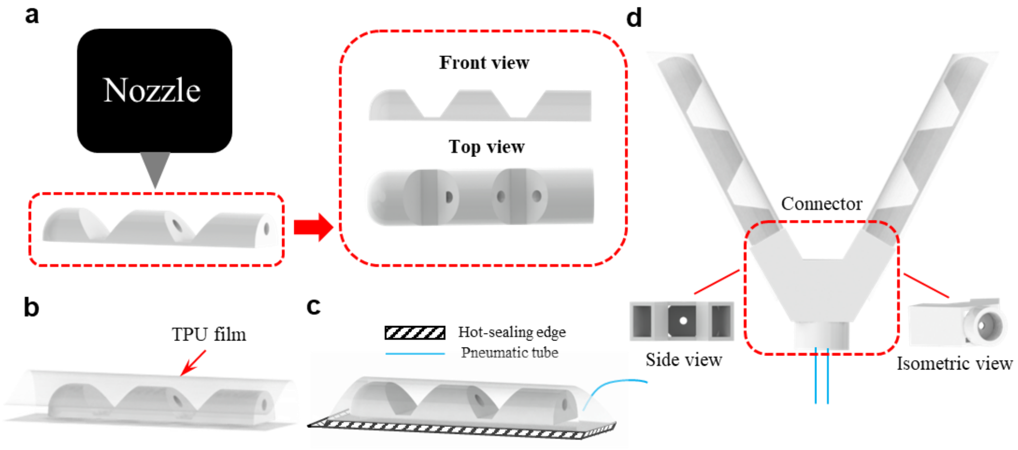

Supplementary Fig. 22. Fabrication process of the vacuum-actuated gripper. a, Thermoplastic polyurethane (TPU, ESUN, Xiaogan Esun New Material Co., Ltd, China) is used to 3D print the soft finger with the printer Raise3D Pro3 (Shanghai Fusion Tech Co., Ltd, China). **b**, A piece of TPU film is used to wrap the finger. **c**, The edges of the TPU film are sealed with a heat-sealing machine (Deli 16499, Deli Group Co., Ltd, China), followed by inserting a pneumatic tube into the TPU film. **d**, Two identical vacuum-actuated fingers are inserted into the connector to form a soft gripper, which has a threaded hole and can connect with the origami module.

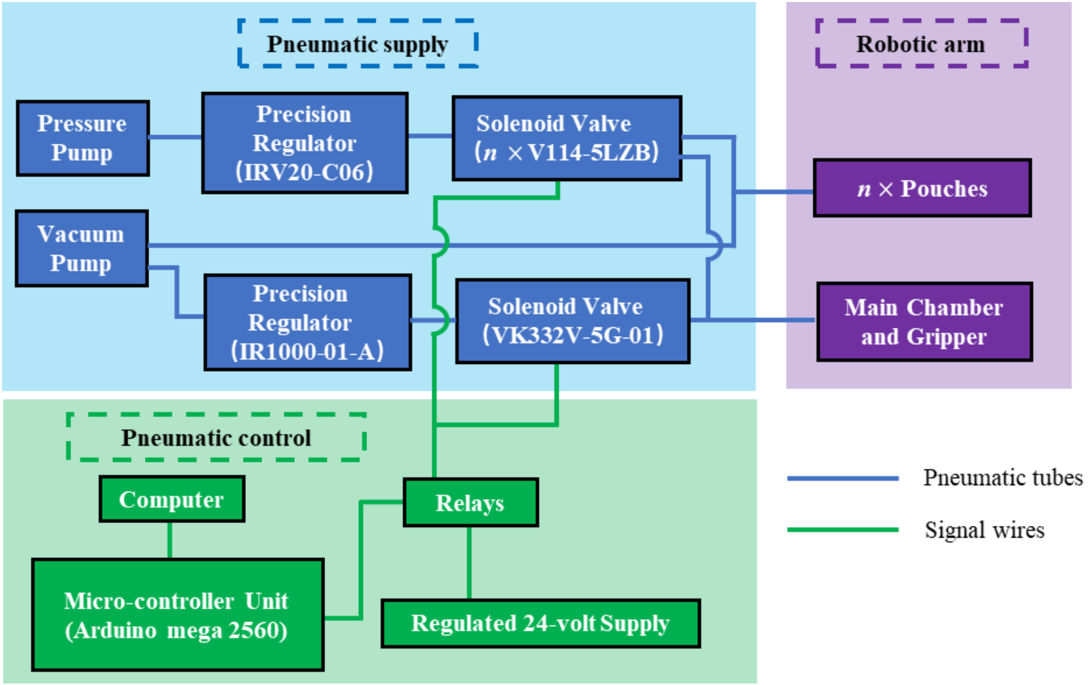

Supplementary Fig. 23. Control system of the three-module/four-module robotic arm.

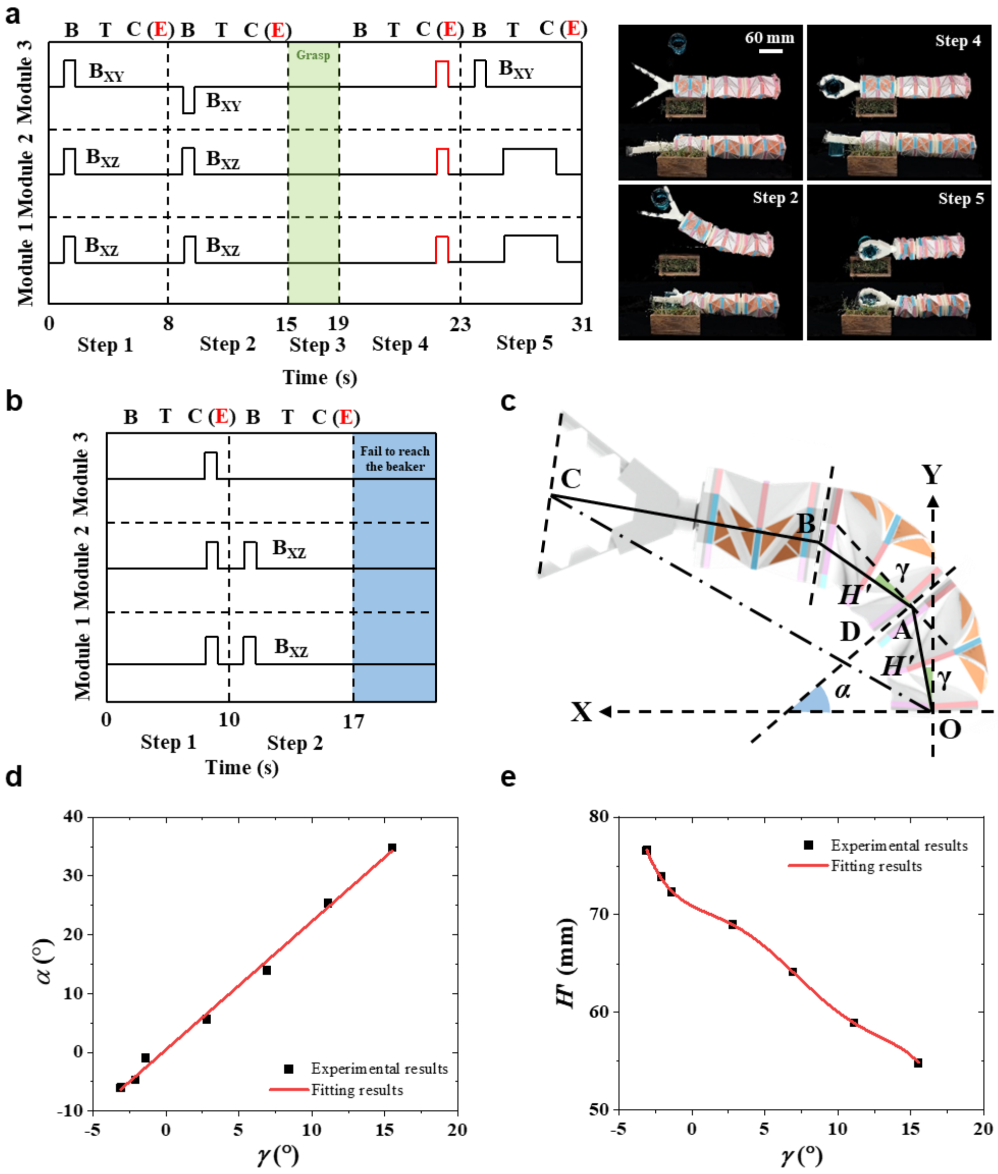

Supplementary Fig. 24. Three-module robotic arm. a, Diagram of the three-module robotic arm's motion modes and experimental results of the three-module robotic arm beaker-grasping and watering grass from top view and front view. **b,** Diagram of the three-module robotic arm's motion modes in **Fig. 4c**. **c,** Diagram of the working boundary calculation of the three-module robotic arm. H' and γ represent the length between the central points of the top and bottom cap, and the angle between the vertical line and the line linked by the central points of the top and bottom cap. **d,** Experimental and fitting results of the relationship between α and γ. **e,** Experimental and fitting results of the relationship between α and H'.

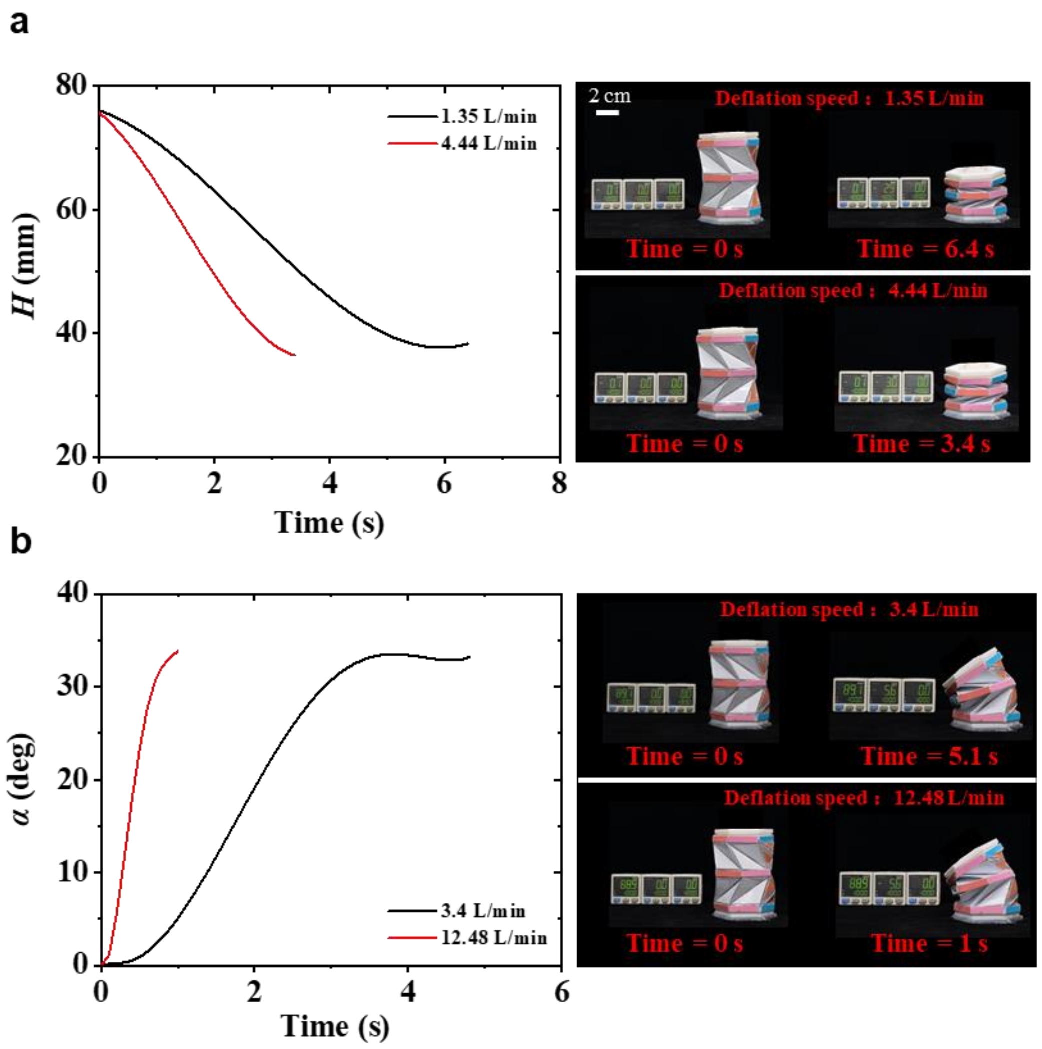

Supplementary Fig. 25. Deformation of a module with different deflation speed in contraction (a) and bending motion (b). The air flow is tested with flowmeter (MF5708, Siargo Co., LTD. Germany). The pressure is tested with gas-pressure meter (DP-101, Panasonic Co., LTD. Japan). The results show that the dynamic process depends on the rate of air flow, but the final states (i.e., deformation modes) do not depend on the rate of air flow.

Supplementary Table S1 Parameters of the **Equation S1** and **Equation S2**

P_1	P_2	P_3	P_4	P_5	P_6	P_7	P_8
-0.0001249	0.004431	-0.04865	0.1393	-0.7961	70.92	2.183	0.452

Supplementary Table S2 Parameters of the **Equation S3** and **Equation S4**

Q_1	Q_2	Q_3	Q_4	Q_5	Q_6
0.0003486	-0.02875	0.3646	-1.455	-1.023	75.54
Q_7	Q_8	Q_9	Q_{10}	Q_{11}	Q_{12}
-0.0002789	-0.002046	0.05436	0.1297	0.5385	-1.281

Supplementary Table S3 Parameters of the **Equation S5** and **Equation S6**

S_1	S_2	S_3	S_4	S_5
-0.0002012	0.009162	-0.1666	1.443	-7.013
S_6	S_7	S_8	S_9	S_{10}
79.81	0.01361	-0.2349	3.164	-2.892
