# Supplementary material for: Plug & play origami modules with all-purpose deformation modes
Source: Nat Commun. 2023 Jul 19;14:4329. doi: 10.1038/s41467-023-39980-7 (PMC10356792; doi:10.1038/s41467-023-39980-7)
Supplement: Supplementary file 3 — Description of Additional Supplementary Files [file 41467_2023_39980_MOESM3_ESM.pdf]

<u>Description of Additional Supplementary Files</u>

Supplementary Movie 1. Twisting-contraction motion of the one-level origami.

Supplementary Movie 2. Bending-twisting motion of the one-level origami.

Supplementary Movie 3. Contraction motion of the origami.

Supplementary Movie 4. Bending motion of the origami.

Supplementary Movie 5. Twisting motion of the origami.

Supplementary Movie 6. Twisting-contraction motion of the origami.

Supplementary Movie 7. Bending-twisting motion of the origami.

Supplementary Movie 8. Bending-contraction motion of the origami.

Supplementary Movie 9. Bending-twisting contraction motion of the origami.

Supplementary Movie 10. Three-module robotic arm: pouring water out of a cup.

Supplementary Movie 11. Three-module robotic arm: watering grass.

Supplementary Movie 12. Three-module robotic arm: failing to reach a distant water cup.

Supplementary Movie 13. Plug & play process.

Supplementary Movie 14. Four-module robotic arm: reaching the cup and pouring water out.

Supplementary Movie 15. Complex motion of the fourmodule robotic arm.

Supplementary Movie 16. Underwater complex motion of the four-module robotic arm.
